# Prevalence of orthorexia nervosa among medical students

**DOI:** 10.1192/j.eurpsy.2024.417

**Published:** 2024-08-27

**Authors:** A. Mellouli, S. Ellouze, M. Barkallah, M. Turki, B. Ben Jmeâa, N. Halouani, J. Aloulou

**Affiliations:** ^1^Psychiatry B, Hedi Chaker University Hospital, Sfax, Tunisia

## Abstract

**Introduction:**

Orthorexia nervosa is defined as an unhealthy obsession with eating healthy food. Recent studies currently demonstrated that students in health-oriented academic programs, highly focused on nutrition and physical exercise, are more prone to develop orthorexia nervosa than students in other educational areas.

**Objectives:**

Determine the prevalence of orthorexia nervosa in medical students and identify associated factors.

**Methods:**

We conducted a cross-sectional, descriptive, and analytical study in the faculty of medicine of Sfax in Tunisia, between February and April 2023. We used ORTO-15 for the assessment of orthorexia.

**Results:**

The research has enrolled 220 students. Their mean age was 21.40±1.68 years, with female predominance (70%). The mean Body mass index (BMI) was 22.46±4.15 kg/m2. The prevalence of overweight (BMI≥25 kg/m2) and obesity (BMI≥30 kg/m2) were respectively 19.5% and 3.6%. Over a third of students (34.1%) were using means of weight control, of which the diet represented 62.66% of cases. The participants had consulted a nutritionist in 11.4% of cases. The ORTO-15 mean total score was 36.88±6.76, with a mean score of 12.95±2.69 for cognitive dimension, 13.31±2.70 for clinical dimension, and 10.61±2.52 for emotional dimension. A total of 60% of participants had a score under the threshold.

Orthorexia was significantly associated with female gender (p<10 ^-3^), overweight or obesity (p=0.037), the use of weight control methods (p<10^-3^), following a diet (p<10^-3^), and consulting a nutritionist (p=0.009).

**Conclusions:**

In our study, orthorexia seems to be quite widespread among medical students, particularly females, who are overweight or obese, and who use weight control methods.

**Disclosure of Interest:**

None Declared

